# Healthcare-Associated Infections: Knowledge Score and Awareness Among Nurses in Hospitals from North-East Romania

**DOI:** 10.3390/healthcare14010044

**Published:** 2025-12-24

**Authors:** Nicoleta Luchian, Cristian Guțu, Alina Pleșea-Condratovici, Camer Salim, Mădălina Irina Ciuhodaru, Liviu Stafie, Mihaela Roxana Popescu, Mădalina Nicoleta Matei, Doina Carina Voinescu, Mădălina Duceac (Covrig), Eva Maria Elkan, Letiția Doina Duceac

**Affiliations:** 1 Doctoral School of Biomedical Sciences, Faculty of Medicine and Pharmacy, “Dunărea de Jos” University of Galați, 47 Domnească Street, 800008 Galati, Romania; nicoletaluchian13@yahoo.com (N.L.); madalinaduceac@yahoo.ro (M.D.); 2 Faculty of Medicine and Pharmacy, “Dunărea de Jos” University of Galați, 47 Domnească Street, 800008 Galati, Romania; dr.c.gutu@gmail.com (C.G.); madalina.matei@ugal.ro (M.N.M.); carinavoinescu@gmail.com (D.C.V.); cojocarumariaeva@yahoo.com (E.M.E.); letimedr@yahoo.com (L.D.D.); 3 Faculty of Medicine, Ovidius University Constanța, 900740 Constanta, Romania; 4 Faculty of Medicine, “Grigore T. Popa” University of Medicine and Pharmacy, 16 Universității Street, 700115 Iasi, Romania; madalina.nour@umfiasi.ro (M.I.C.); liviu.stafie@umfiasi.ro (L.S.); 5 Faculty of Dental Medicine, “Grigore T. Popa” University of Medicine and Pharmacy, 16 Universității Street, 700115 Iasi, Romania; mihaela.popescu@umfiasi.ro

**Keywords:** healthcare-associated infections, epidemiology, prevention, risk factors, nurses

## Abstract

*Background and Objectives*: Healthcare-associated infections (HAIs) are a major cause of morbidity and mortality and can lead to serious long-term consequences, increased hospital length stay, higher rates of antibiotic resistance, and additional financial costs. The study aim was to highlight important aspects related to the level of knowledge of HAIs, risk factors for HAI and methods of preventing HAIs among nurses from urban hospitals in the North-East of Romania. *Materials and Methods*: We conducted a cross-sectional study on the level of knowledge of HAI problems among medical personnel (nurses). The study group consisted of 288 nurses who responded online to a questionnaire. *Results*: Multivariate analysis suggested that 45.5% of the value of the knowledge score of prevention and limitation of HAIs could be determined by the answers to the questions regarding the importance of the nurse role, professional experience, and training. Moreover 84.6% of the knowledge score could be determined by the answers to the questions regarding the assessment of the importance of factors related to lack of medical personnel, professional burnout, insufficient knowledge and staff training, inefficient team, poor collaboration within departments, stressful work environment, staff health status, multiple tasks at work (*p* = 0.038). While 50% of the knowledge score could be explained by responses regarding personal problems related to daily activity; physical and mental health status; and physical, psychological, and social components (*p* = 0.038). *Conclusions*: Our study highlighted aspects of the level of knowledge regarding HAIs among nurses, an issue that plays a key role in hospital management. Overall, the knowledge score for the prevention and limitation of HAIs was higher in nurses/females aged over 40 and in nurses with more than 15 years of experience.

## 1. Introduction

Healthcare-associated infections (HAIs) represent a persistent challenge to healthcare systems globally, and Europe is no exception. Despite ongoing efforts to control these infections, the burden remains high, leading to approximately 37,000 deaths each year in Europe and worldwide [1].

The prevalence of HAIs and the incidence of antimicrobial resistance have been closely monitored through point prevalence surveys conducted in the European Union/EEA. These surveys have provided valuable information on the current status of HAIs in Europe, including types of infections, risk factors, and the effectiveness of prevention and control measures [2,3].

HAIs are a major cause of morbidity and mortality and can lead to serious long-term consequences, increased length of hospital stay, increased rates of antibiotic resistance, additional financial costs and even preventable deaths [4,5].

More recent point prevalence studies conducted between 2016 and 2017 have further expanded our understanding of the HAIs landscape in Europe. These studies involved over 400,000 patients in acute care hospitals and long-term care facilities in 28 countries, providing a comprehensive assessment of the prevalence of these infections and the associated antimicrobial resistance patterns [6].

The most frequently reported organisms were *Escherichia coli*, *Staphylococcus aureus,* and norovirus. The incidence of HAIs was higher in the elderly and in emergency cases. There was an increase in the rate of HAIs in the summer months (pneumonia, respiratory infections, surgical site, and gastrointestinal infections) and during the winter, gastrointestinal infection with norovirus. The specialties with the highest incidence were intensive care, urology, and cardiothoracic surgery [7,8,9,10,11,12,13].

In Romania, according to reports, the incidence of HAIs has varied between 1995 and 2020, as well as during the COVID-19 pandemic and in the years that followed. Although there are annual fluctuations, the general trend indicates an increase in the reporting of these infections, which may reflect both a real increase in incidence and an improvement in surveillance and reporting systems. It is important to note that data on the incidence of HAIs may vary depending on the country and the reporting methodology. Continued prevention and control efforts, including the implementation of hygiene programs, the judicious use of antibiotics, and strict infection monitoring, remain essential to reduce the incidence of HAIs in Europe [14,15,16,17].

The European Union has implemented several initiatives to combat AMR, including the European Infection Control Strategy and the Action Plan on Combating Antimicrobial Resistance. ECDC and Reinforcement of Training for European Countries support the exchange of information and good practices between member countries [15]. AMR remain a major challenge in Europe, requiring continued efforts from healthcare professionals, authorities, and patients. By adhering to hygiene standards and responsible use of antibiotics and implementing prevention strategies, the impact of these infections can be significantly reduced, contributing to the safety and efficiency of the European healthcare system [18,19,20,21,22].

HAIs have been identified as a significant challenge in terms of patient safety. The CDC has viewed HAIs as a significant threat to patient safety in hospitals and a burden on health systems and the community. Decades after the publication of a report stating the well-known fact—To Err is Human (no one is perfect, the phrase that referred to the report of nosocomial infections in the 2000s, in the USA), the CDC implemented programs and strategies to eliminate HAIs, in the early 2020s but was limited by the COVID-19 pandemic [23].

Another problem that other authors in the USA [24] and Europe [25] have also reported and that hospitals in our region are also facing is related to the lack of staff. This fact represents a burden for hospitals, in order to be able to provide high-quality services and maintain low HAIs rates. Infection control in healthcare facilities relies on professional staff and specialized teams and is mandatory, with regulations and protocols similar to hospitals [26]. This lack of healthcare personnel can have several negative consequences, both for themselves and for patients, such as increased time pressure and work effort and lower compliance with patient safety principles leading to a lower quality of care and, therefore, a higher risk of HAIs. Limited work experience among nurses can also be a risk factor for patient safety. The patient’s risk of having a HAI could be further increased if insufficient staffing is combined with limited work experience because more work experience may be required to prioritize tasks if the workload is higher; however, to date, few studies have been conducted on this topic [27,28,29].

The study aim was to highlight important aspects related to the level of knowledge of HAIs, risk factors for HAIs, and methods of preventing HAIs among nurses working in hospitals in North-East Romania. The study objectives: to evaluate the nurses’ awareness of risk factors associated with HAIs; to identify the main gaps in knowledge related to HAIs prevention and control measures; and to explore associations between demographic characteristics and knowledge score.

## 2. Materials and Methods

### 2.1. Study Design

We conducted a cross-sectional study on the level of knowledge of HAI problems among nurses.

### 2.2. Study Settings

The study was conducted in hospitals in the North-East region of Romania, which serve both urban and rural populations (“Prof. Dr. N. Oblu” Clinical Emergency Neurosurgery Hospital, Iași and Municipal Emergency Hospital, Pașcani, Iași County), during the year 2024.

### 2.3. Study Population

The study group consisted of 288 subjects, mid-level medical staff (nurses) who responded online to the following questionnaire: Questionnaire for the evaluation of the level of information regarding healthcare-associated infections among medical professionals (nurses) See Supplementary Materials.

Inclusion criteria for the study: registered nurses with at least one year of professional experience, currently employed in hospital wards (surgical, medical, or intensive care unit) within hospitals in North-East Romania.

Exclusion criteria from the study: physicians, nursing students, administrative staff, and personnel not directly involved in patient care.

The selection method of the study group was non-probability sampling, choosing a convenience sample based on predetermined specific criteria (those listed above). After applying the inclusion and exclusion criteria, we obtained a representative study group suitable for the purpose of our research.

Participants’ contact details were obtained through official collaboration with the hospital management teams. Invitations to participate in the study were distributed at in-person meetings facilitated by each hospital’s medical staff department. No personal contact data were accessed without prior institutional approval, and confidentiality was maintained throughout the recruitment process in accordance with ethical guidelines.

### 2.4. The Applied Questionnaire

The questionnaire was distributed via an online link (in google form) to medical professionals (nurses) from the city hospitals assigned to the region. They were informed about the purpose of the study, the confidentiality of the data, and how to respond to the questionnaire voluntarily and anonymously.

#### 2.4.1. Construction of Our Own Questionnaire

We developed our own questionnaire, consisting of 36 items, based on information regarding HAIs published on the official websites of the WHO, CDC, ECDC, and of the Romanian Ministry of Health, as well as studies on the topic of HAIs [6,17,21].

The items were written in Romanian, without any subsequent translation. The questionnaire was initially applied to a small group of medical professionals, to verify that the questions are intelligible and clear and the answer options have the correct perceived meaning. We subsequently checked and modified the items that were unclear. The questions were MCQ (multiple-choice questions) and OEQ (open-ended questions). The number of questions, although substantial, was chosen at 36 to encompass as wide a range as possible from the vast issues of HAIs.

The data were collected using the online questionnaire method in google form, during the year 2024.

#### 2.4.2. Questionnaire Validation

The database was initially completed in MS Excel, also using google forms, with their subsequent completion and improvement. The Cronbach alpha value = 0.818 based on the standardized items of the importance of professional experience in preventing and limiting HAIs, identifying risk factors and the measures applied represent good values in relation to the necessary threshold (0.700) for validating the application of this questionnaire.

### 2.5. Statistical Processing

The chi-squared test or Kruskal–Wallis test are non-parametric tests that compare 2 or more frequency distributions from the same population. The Skewness or Kurtosis tests (−2 < *p* < 2) are tests that measure the normality of the value series, in order to determine whether the variables are continuous or not. The data were loaded and processed using the statistical functions in SPSS 18.0 at the 95% significance level. The lower the *p*-value is compared to this value, the stronger the significance.

The principle of scores was applied to all subjects (the same questionnaire) and is calculated according to the specifications in Table 1.

### 2.6. Scientific Research Ethics and GenAI Using

Formal approval for the study was obtained from the Hospital Ethics Committee and the general provisions of the Declaration of Helsinki on Medical Research Involving Human Subjects were followed.

The data obtained through the questionnaire are anonymous and the responses are confidential. The study respected all principles of medical ethics. Informed consent was obtained before completing the questionnaire.

The study was approved by the Ethics Committee of “Prof. Dr. N. Oblu” Clinical Emergency Neurosurgery Hospital (number of approval 408/24.02.2023), Ethics Committee of Municipal Emergency Hospital of Pașcani (number of approval 90/03.01.2024), and University Ethics Committee (CEU) of the “Dunărea de Jos” University of Galați, Romania (number of approval: 83/CEU/27.11.2024).

We did not use GenAI to generate text, data, or graphics, nor to assist in study design, data collection, analysis, interpretation, or text editing.

## 3. Results

### 3.1. Demographic Characteristics

The age of the subjects who responded to the questionnaire revealed a higher frequency between 41 and 60 years. Of the total study group, only 6.6% of the subjects were male, the gender ratio being F/M = 14.2/1. Among males, 21.1% were under 30 years of age, while among females only 7.1%. Ages over 60 were recorded with reduced frequencies in both sexes: 5.3% in males and 0.7% in females (*p* = 0.106).

**Figure 1:**
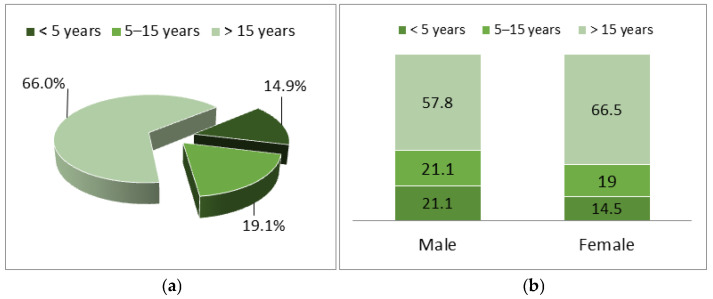
Study group structure (**a**) age groups and (**b**) length of service.

Of the total study group, 14.9% of the subjects had less than 5 years of work experience, more frequently in men (21.1% vs. 14.5%), and 66% had more than 15 years of experience, more frequently in women (57.9% vs. 66.5%), but the percentage distributions were not statistically significant (*p* = 0.703). (Figure 1, Table 2)

### 3.2. Multivariate Analysis Models

Multivariate analysis, through linear regression suggests that 45.5% of the value of the knowledge score of prevention and limitation of HAIs can be determined by the answers to the questions regarding the importance of the role of the nurse (R1), professional experience (R2), and professional training (R3) in the prevention and limitation of HAIs. Factors related to the Unit, Staff, and Patient (adjusted R-squared = 0.455; sig *p* = 0.038) (Table 3).

Multivariate analysis suggested that 84.6% of the value of the knowledge score on prevention and limitation of HAIs could be determined by the answers to the questions regarding the assessment of the importance of factors related to staff shortage (P1), professional exhaustion/burnout (P2), insufficient knowledge (P3), inefficient team (P4), poor collaboration with the HAIs Prevention and Control Department (P5), poor staff training (P6), stressful work environment (P7), staff health status (P8), and multiple tasks at work (P9) (adjusted R-squared = 0.846; sig *p* = 0.038) (Table 4). Table S1 in Supplementary Materials.

Multivariate analysis suggested that approximately 50% of the value of the knowledge score on prevention and limitation of HAIs could be determined by the answers to the questions regarding personal problems in relation to daily activity that can influence HAIs prevention: personal health status (S1), personal physical and mental health status compared to the previous year (S2), influence of health problems on daily activities—physical component (S3), mental component (S4), social component (S5)—hospitalizations in the last 5 years (S6), professional exhaustion in the last year (S7), body pain in the last time (S8), and work affected due to health problems (S9) (adjusted R-squared = −0.846; sig *p* = 0.038) (Table 5) (Tables S2 and S3 in Supplementary Materials.)

**Figure 2:**
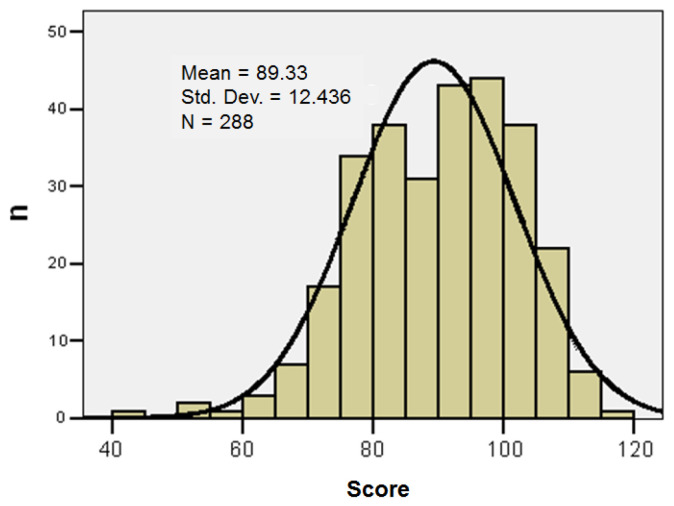
Histogram of the knowledge score of prevention and limitation of HAIs (Std. Dev.—Standard Deviation).

The series of values for the knowledge score of prevention and limitation of HAIs was homogeneous, which suggests that statistical significance tests can be applied: variations in the range 43–118; group mean 89.33 ± 12.44; median 91; Skewness test result *p* = −0.473. (Table 6, Figure 2)

The knowledge score of prevention and limitation of HAIs varied in the range of 43–118. A low score suggests a “worse” state, translating into a lower degree of knowledge of prevention and limitation of HAIs, and a high score suggests a “better” state, translating into a higher degree of knowledge of prevention and limitation of HAIs. Taking into account these results, the score was standardized into three classes, as presented in Table 7.

The knowledge score of prevention and limitation of HAIs was higher in females (91%; *p* = 0.292), in ages over 40 (68.7%; *p* = 0.753), and in personnel with more than 15 years of work experience (62.7%; *p* = 0.196) (Table 8).

We calculated the correlation between the knowledge score of prevention and limitation of HAIs and the answers regarding the level of knowledge in prevention and limitation of HAIs. (Table S4 in Supplementary Materials).

Personal problems could greatly influence the prevention of HAIs; the responses highlighted their correlation with a high knowledge score. (Table S5 in Supplementary Materials).

## 4. Discussion

The level of knowledge among healthcare personnel regarding HAIs has a direct impact on the prevention and control of these infections. Education and awareness are essential for improving infection control measures in healthcare facilities. The use of modern technology, promoting a culture of safety, and creating a culture in which HAIs prevention is a priority and healthcare professionals feel supported in their efforts to reduce HAIs, as well as encouraging teamwork between medical, nursing, and cleaning staff to maintain a clean and safe environment, can be some ideas derived from the application of our questionnaire.

Regarding the level of knowledge in the prevention and limitation of HAIs, the answers “very much” to the following questions highlighted a high knowledge score, as follows: a very large nurse role (97%; *p* = 0.039); professional experience (88.1%; *p* = 0.001); professional training (95.5%; *p* = 0.007); know the influence of Unit-related factors (52.2%; *p* = 0.001); know the influence of Staff-related factors (52.2%; *p* = 0.001); know the influence of Patient-related factors (49.3%; *p* = 0.001); recognize the influence of staff shortages (61.2%; *p* = 0.001); recognize the influence of professional burnout (74.6%; *p* = 0.001); recognize the influence of insufficient knowledge (73.1%; *p* = 0.001); inefficient collaboration with the HAIs Department of Prevention and Control (62.7%; *p* = 0.001); recognize the influence of poor staff training (73.1%; *p* = 0.001); stressful work environment (76.1%; *p* = 0.001); staff health status (79.1%; *p* = 0.001); and multiple work tasks (82.1%; *p* = 0.001).

Other research has also highlighted that the interaction between coping mechanisms, lifestyle habits, and perceived support systems play a crucial role in determining the resilience of healthcare workers to such stressors. Adaptive coping strategies, healthy lifestyle behaviors, and robust social and organizational support systems are essential for maintaining psychological well-being [30,31].

Our study highlighted that personal problems of healthcare professionals could influence the level of reaction and the ability to recognize and prevent HAIs. Thus, the responses highlighted certain factors related to health status, which showed correlation with a high knowledge score, such as good health status (61.2%; *p* = 0.031); health status the same as last year (52.2%; *p* = 0.032); physical component of health status (59.7%; *p* = 0.001); mental health (73.1%; *p* = 0.001); social health (41.8%; *p* = 0.001); and health problems (41.8%; *p* = 0.004).

Other studies have also highlighted that health professionals should be prepared to deal with complex systems to ensure the best interests of patients. Every healthcare professional should have the necessary knowledge and skills to be able to react correctly in order to prevent and control HAIs. However, healthcare personnel are faced with complex situations in which resources are inadequate and their work is overwhelmed by other demands, including personal health-related problems [32,33].

Another problem that we also face at the hospital level is that medical staff may consider that the prevention of HAIs does not have such an important role in hospital management [34], compared to their clinical role, and adherence to infection prevention and control practices is sometimes influenced by numerous factors. Therefore, we believe that hospitals should frequently and regularly carry out education and training programs on the prevention and control of HAIs, programs in which all medical and auxiliary staff should participate. Studies worldwide have framed these practices as an institutional culture of safety, that is, a safe hospital environment, with the aim of the risk of HAIs being reduced as much as possible [35].

Published research, as well as hospital practice, has shown that nurses play a central role in implementing HAIs prevention and control measures, as nurses provide direct patient care and implement HAIs prevention and control protocols. Thus, it is important to ensure, through hospital management, the continuous training of these healthcare professionals, with access to the latest prevention techniques and technologies [36].

As our study highlights, continuing education of healthcare professionals is important in understanding risk factors and prevention methods for HAIs.

Knowledge gaps and challenges could be related to a lack of continuing education or irregular training on best practices in HAI prevention and control. Also, misconceptions or outdated concepts prevent challenges, as well as the existence of barriers related to institutional culture that may not prioritize HAIs prevention practices or limit human and material resources [37].

A study conducted in India on a group of nursing students showed that in the hospitals where the questionnaire was administered, insufficient attention was paid to explaining the benefits of prevention mechanisms and the consequences of HAIs. The authors noted that recognizing knowledge gaps is essential to improving prevention practices [38].

A hospital, an even a smaller one, that has a strong safety culture will promote education, encourage communication and interdisciplinarity, and foster a positive, proactive, and collaborative climate among healthcare professionals to improve HAI prevention and control practices, which will definitely lead to improved patient care. Based on our questionnaire and multivariate analysis models, we can conclude that improving healthcare staff knowledge of HAIs is crucial in preventing these infections and reducing their impact on patients. Continuing education, effective training programs, and a strong institutional commitment to infection control are essential to reducing HAIs and improving patient outcomes.

Burnout and work—life balance. Recent studies have highlighted the importance of a balance between professional and personal life, with the effective harmonization of professional and family responsibilities [39]. A large cross-sectional study, carried out in 50 hospitals in China, showed that over 60% of the nurses interviewed reported an increased level of anxiety and professional burnout, and over 50% suffered varying degrees of stress and emotional exhaustion [40]. In this direction, we consider that our study also makes an important contribution, highlighting the risk factors and extra-professional problems of medical staff, as well as the exhaustion to which they are subjected, through daily stress and shifts related to the hospital schedule [41,42,43].

Study limitations. The study utilized an online questionnaire to collect data. It is possible that the participants who chose to complete the survey were more motivated or interested in the topic, which could have influenced the results. Additionally, the questionnaire was relatively lengthy and difficult to complete, which may have caused some respondents to abandon it.

Future research should involve a larger sample size and control for key variables, and adapt a mixed-methods approach to gain a broader range of perspectives. It should also take into account the specifics of each hospital to produce more generalizable results.

## 5. Conclusions

Our study highlighted aspects of the level of knowledge regarding HAIs among medical staff (nurses), an issue with an important key role in hospital management, as well as increasing the awareness of nurses regarding HAIs. This could improve HAIs prevention strategies and their application in practice, which ensure access to health services. This research provided a more accurate understanding of the level of knowledge of HAIs among mid-level medical staff, as indicated by the knowledge score, highlighting the gaps and areas that need to be reviewed and improved. This is essential for quality management in hospitals and the effective management of HAIs.

## Figures and Tables

**Table 1:** Principles of calculating scores for the applied questionnaire.

Scoring Principle	Response Elaboration
For each subject the score is the sum of the answers to each of the questions regarding the level of knowledge about the prevention and limitation of HAIs:	1 yes 0 no 1 not at all 2 a little 3 moderately 4 quite a bit 5 very much
For questions regarding health status:	1 much worse 2 worse 3 deficient 4 much better 5 excellent
The primary score is standardized as follows:	a low score is interpreted as “worse” a high score is interpreted as “better”

**Table 2:** Demographic and professional data.

Statistical Variables	Number (*n* = 288)	%
Distribution by age group		
18–30 years	23	8.1
31–40 years	56	19.4
41–60 years	206	71.5
61+ years	3	1
Distribution by gender		
female	243	84.4
male	45	15.6
Length of service		
<5 years	55	19.1
5–15 years	190	66.0
>15 years	43	14.9

**Table 3:** Multivariate linear regression. Dependent variable Score1. Independent variables: the answers to the questions regarding the importance of the role of the nurse (R1), professional experience (R2), and professional training (R3) in the prevention and limitation of HAIs. Factors related to the Unit, Personnel, and Patients.

Model	R	R-Squared	Adjusted R-Squared	Estimated Standard Error	Statistical Test				
					R-Squared Change	F Change	df1	df2	Sig. F Change
1	0.293 (a) *	0.086	0.083	11.910	0.086	26.927	1	286	0.000
2	0.330 (b)	0.109	0.103	11.780	0.023	7.320	1	285	0.007
3	0.339 (c)	0.115	0.105	11.764	0.006	1.821	1	284	0.178
4	0.644 (d)	0.414	0.406	9.586	0.300	144.674	1	283	0.000
5	0.677 (e)	0.459	0.449	9.231	0.045	23.192	1	282	0.000
6	0.683 (f)	0.467	0.455	9.177	0.008	4.325	1	281	0.038

* a—Predictors: (Constant), R1; b—Predictors: (Constant), R1,R2; c—Predictors: (Constant), R1, R2, R3; d—Predictors: (Constant), R1, R2, R3, Unit; e—Predictors: (Constant), R1, R2, R3, Unit, Personnel; f—Predictors: (Constant), R1, R2, R3, Unit, Personnel, Patient.

**Table 4:** Multivariate linear regression. Dependent variable Score2. Independent variables: answers to questions regarding the assessment of the importance of factors related to staff shortage (P1), professional exhaustion/burnout (P2), insufficient knowledge (P3), inefficient team (P4), poor collaboration with the HAIs Prevention and Control Department (P5), poor staff training (P6), stressful work environment (P7), staff health status (P8), and multiple tasks at work (P9).

Model	R	R-Squared	Adjusted R-Squared	Estimated Standard Error	Statistical Test				
					R-Squared Change	F Change	df1	df2	Sig. F Change
1	0.618 (a) *	0.382	0.380	9.790	0.382	177.123	1	286	0.001
2	0.703 (b)	0.494	0.491	8.873	0.112	63.170	1	285	0.001
3	0.855 (c)	0.732	0.729	6.477	0.237	250.892	1	284	0.001
4	0.880 (d)	0.774	0.770	5.960	0.042	52.381	1	283	0.001
5	0.897 (e)	0.804	0.801	5.554	0.030	43.881	1	282	0.001
6	0.899 (f)	0.808	0.804	5.510	0.004	5.511	1	281	0.020
7	0.910 (g)	0.829	0.825	5.207	0.021	34.707	1	280	0.001
8	0.919 (h)	0.844	0.839	4.984	0.015	26.594	1	279	0.001
9	0.922 (i)	0.851	0.846	4.884	0.007	12.521	1	278	0.001

* a—Predictors: (Constant), P1; b—Predictors: (Constant), P1, P2; c—Predictors: (Constant), P1, P2, P3; d—Predictors: (Constant), P1, P2, P3, P4; e—Predictors: (Constant), P1, P2, P3, P4, P5; f—Predictors: (Constant), P1, P2, P3, P4, P5, P6; g—Predictors: (Constant), P1, P2, P3, P4, P5, P6, P7; h—Predictors: (Constant), P1, P2, P3, P4, P5, P6, P7, P8; i—Predictors: (Constant), P1, P2, P3, P4, P5, P6, P7, P8, P9.

**Table 5:** Multivariate linear regression. Dependent variable Score 3. Independent variables are the answers to the questions regarding the assessment of the importance of factors related to personal health status (S1), personal physical and mental health status compared to the previous year (S2), influence of health problems on daily activities—physical component (S3), mental component (S4), and social component (S5)—hospitalizations in the last 5 years (S6), professional exhaustion in the last year (S7), body pains recently (S8), and work affected due to health problems (S9).

Model	R	R-Squared	Adjusted R-Squared	Estimated Standard Error	Statistical Test				
					R-Squared Change	F Change	df1	df2	Sig. F Change
1	0.194 (a) *	0.038	0.034	12.222	0.038	11.143	1	286	0.001
2	0.200 (b)	0.040	0.033	12.227	0.003	0.759	1	285	0.384
3	0.683 (c)	0.466	0.460	9.136	0.426	26.520	1	284	0.000
4	0.697 (d)	0.486	0.479	8.978	0.020	11.074	1	283	0.001
5	0.703 (e)	0.494	0.485	8.928	0.008	4.199	1	282	0.041
6	0.711 (f)	0.506	0.495	8.834	0.012	6.999	1	281	0.009
7	0.715 (g)	0.512	0.500	8.798	0.006	3.335	1	280	0.069
8	0.717 (h)	0.514	0.500	8.795	0.002	1.143	1	279	0.286
9	0.719 (i)	0.517	0.501	8.784	0.003	1.718	1	278	0.191

* a—Predictors: (Constant), S1; b—Predictors: (Constant), S1, S2; c—Predictors: (Constant), S1, S2, S3; d—Predictors: (Constant), S1, S2, S3, S4; e—Predictors: (Constant), S1, S2, S3, S4, S5; f—Predictors: (Constant), S1, S2, S3, S4, S5, S6; g—Predictors: (Constant), S1, S2, S3, S4, S5, S6, S7; h—Predictors: (Constant), S1, S2, S3, S4, S5, S6, S7, S8; i—Predictors: (Constant), S1, S2, S3, S4, S5, S6, S7, S8, S9.

**Table 6:** Descriptive statistical indicators of the knowledge score of prevention and limitation of HAIs.

N		288
Mean		89.33
Median		91
Standard Deviation		12.44
Variance		13.93
Skewness Test		−0.473
Standard Error Skewness		0.144
Minimum		43
Maximum		118
Percentile	25	80
	50	91
	75	99

**Table 7:** Group structure according to the knowledge score of prevention and limitation of HAIs.

Score	n	%
Low	65	22.6
Moderate	156	54.2
High	67	23.3

**Table 8:** Correlation between the knowledge score of prevention and limitation of HAIs and the demographic characteristics of the study group.

Items/Demographics	Low Score (n = 65)		Moderate Score (n = 156)		High Score (n = 67)		Chi-Squared Test (*p*)
	n	%	n	%	n	%	
Female	59	90.8	149	95.5	61	91.0	0.292
Age > 40 years	48	73.8	115	73.7	46	68.7	0.753
Length of service > 15 years	47	72.3	101	64.7	42	62.7	0.196

## Data Availability

The original contributions presented in this study are included in the article/Supplementary Materials. Further inquiries can be directed to the corresponding authors.

## Supplementary Materials

The following supporting information can be downloaded at: https://www.mdpi.com/article/10.3390/healthcare14010044/s1, Table S1. Multivariate linear regression. Regression line coefficients. Model 6 of the role and nurses professional experience in preventing and limiting HAIs; Table S2. Multivariate linear regression. Regression line coefficients. Model 9 assessment of factors favoring the occurrence of HAIs; Table S3. Multivariate linear regression. Regression line coefficients. Model 9 assessment of factors favoring the occurrence of HAIs; Table S4. Correlation between the knowledge score of prevention and limitation of HAIs and the answers regarding the level of knowledge in prevention and limitation of HAIs; Table S5. Correlation between the knowledge score of HAIs prevention and limitation and personal problems that can influence the prevention of HAIs.

## Abbreviations

The following abbreviations are used in this manuscript:

HAI	Healthcare-associated infection
AMR	Antimicrobial resistance
CDC	Centers for Disease Prevention and Control
ECDC	European Centre for Disease Prevention and Control

## References

[1] Zingg W., Holmes A., Dettenkofer M., Goetting T., Secci F., Clack L., Allegranzi B., Magiorakos A.P., Pittet D. (2015). systematic review and evidence-based guidance on organization of hospital infection control programmes (SIGHT) study group. Hospital organisation, management, and structure for prevention of health-care-associated infection: A systematic review and expert consensus. Lancet Infect. Dis..

[2] Viti F., Cartocci A., Perinti R., Guarducci G., Nante N. (2025). Analysis and Impact of Infection Prevention Procedures in Long-Term Care Facilities. J. Prev. Med. Hyg..

[3] Cristina M.L., Spagnolo A.M., Giribone L., Demartini A., Sartini M. (2021). Epidemiology and Prevention of Healthcare-Associated Infections in Geriatric Patients: A Narrative Review. Int. J. Environ. Res. Public Health.

[4] Alrebish S.A., Yusufoglu H.S., Alotibi R.F., Abdulkhalik N.S., Ahmed N.J., Khan A.H. (2023). Epidemiology of Healthcare-Associated Infections and Adherence to the HAI Prevention Strategies. Healthcare.

[5] Duceac L.D., Stafie L., Păvăleanu I., Mitrea G., Baciu G., Banu E.A., Romila L., Luca A.C. (2018). Sepsis in Paediatrics—A Special Form of Infection Associated to Medical Assistance. Int. J. Med. Dent..

[6] Suetens C., Latour K., Kärki T., Ricchizzi E., Kinross P., Moro M.L., Jans B., Hopkins S., Hansen S., Lyytikäinen O. (2018). Prevalence of healthcare-associated infections, estimated incidence and composite antimicrobial resistance index in acute care hospitals and long-term care facilities: Results from two European point prevalence surveys, 2016 to 2017. Eurosurveillance.

[7] Alqurashi M.S., Sawan A.A., Berekaa M.M., Hunasemarada B.C., Al Shubbar M.D., Al Qunais A.A., Huldar A.S., Bojabara L.M., Alamro S.A., El-Badry A.A. (2025). Hospital Hygiene Paradox: MRSA and Enterobacteriaceae Colonization Among Cleaning Staff in a Tertiary Hospital in Saudi Arabia. Medicina.

[8] Stewart S., Robertson C., Pan J., Kennedy S., Dancer S., Haahr L., Manoukian S., Mason H., Kavanagh K., Cook B. (2021). Epidemiology of Healthcare-Associated Infection Reported from a Hospital-Wide Incidence Study: Considerations for Infection Prevention and Control Planning. J. Hosp. Infect..

[9] Zahari N.I.N., Engku Abd Rahman E.N.S., Irekeola A.A., Ahmed N., Rabaan A.A., Alotaibi J., Alqahtani S.A., Halawi M.Y., Alamri I.A., Almogbel M.S. (2023). A Review of the Resistance Mechanisms for β-Lactams, Macrolides and Fluoroquinolones among Streptococcus pneumoniae. Medicina.

[10] Alhazmi A.H., Alameer K.M., Abuageelah B.M., Alharbi R.H., Mobarki M., Musawi S., Haddad M., Matabi A., Dhayhi N. (2023). Epidemiology and Antimicrobial Resistance Patterns of Urinary Tract Infections: A Cross-Sectional Study from Southwestern Saudi Arabia. Medicina.

[11] Alamer A., Alharbi F., Aldhilan A., Almushayti Z., Alghofaily K., Elbehiry A., Abalkhail A. (2022). Healthcare-Associated Infections (HAIs): Challenges and Measures Taken by the Radiology Department to Control Infection Transmission. Vaccines.

[12] Fonseca F., Forrester M., Advinha A.M., Coutinho A., Landeira N., Pereira M. (2024). *Clostridioides difficile* Infection in Hospitalized Patients—A Retrospective Epidemiological Study. Healthcare.

[13] Merza M.A., Mohammed S.A., Qasim A.M., Abdulah D.M. (2023). Sero-prevalence and associated risk factors of blood-borne viral infection among healthcare workers of a tertiary referral hospital: A single-center experience. Health Probl. Civ..

[14] European Centre for Disease Prevention Control (2024). Healthcare-associated infections acquired in intensive care units. Annual Epidemiological Report for 2021.

[15] Coman A., Pop D., Muresan F., Oprescu F., Fjaagesund S. (2025). Surveillance and Reporting of Hospital-Associated Infections—A Document Analysis of Romanian Healthcare Legislation Evolution over 20 Years. Healthcare.

[16] Olaru I., Stefanache A., Gutu C., Lungu I.I., Mihai C., Grierosu C., Calin G., Marcu C., Ciuhodaru T. (2024). Combating Bacterial Resistance by Polymers and Antibiotic Composites. Polymers.

[17] Luchian N., Olaru I., Pleșea-Condratovici A., Duceac M., Mătăsaru M., Dabija M.G., Elkan E.M., Dabija V.A., Eva L., Duceac L.D. (2025). Clinical and Epidemiological Aspects on Healthcare-Associated Infections with *Acinetobacter* spp. in a Neurosurgery Hospital in North-East Romania. Medicina.

[18] Haque M., Sartelli M., McKimm J., Abu Bakar M. (2018). Health care-associated infections—An overview. Infect. Drug Resist..

[19] Freitas J., Lomba A., Sousa S., Gonçalves V., Brois P., Nunes E., Veloso I., Peres D., Alves P. (2025). Consensus-Based Guidelines for Best Practices in the Selection and Use of Examination Gloves in Healthcare Settings. Nurs. Rep..

[20] Luchian N., Salim C., Condratovici A.P., Marcu C., Buzea C.G., Matei M.N., Dinu C.A., Duceac M., Elkan E.M., Rusu D.I. (2025). Episode- and Hospital-Level Modeling of Pan-Resistant Healthcare-Associated Infections (2020–2024) Using TabTransformer and Attention-Based LSTM Forecasting. Diagnostics.

[21] World Health Organization (2022). Global Antimicrobial Resistance and Use Surveillance System (GLASS) Report 2022.

[22] Sartelli M., Marini C.P., McNelis J., Coccolini F., Rizzo C., Labricciosa F.M., Petrone P. (2024). Preventing and Controlling Healthcare-Associated Infections: The First Principle of Every Antimicrobial Stewardship Program in Hospital Settings. Antibiotics.

[23] Croskerry P. (2010). To err is human--and let's not forget it. CMAJ.

[24] Upadhyay S., Smith D.G. (2023). Healthcare Associated Infections, Nurse Staffing, and Financial Performance. Inquiry.

[25] Peutere L., Terho K., Pentti J., Ropponen A., Kivimäki M., Härmä M., Krutova O., Ervasti J., Koskinen A., Virtanen M. (2023). Nurse Staffing Level, Length of Work Experience, and Risk of Health Care-Associated Infections Among Hospital Patients: A Prospective Record Linkage Study. Med. Care.

[26] Baranowska-Tateno K., Micek A., Gniadek A., Wójkowska-Mach J., Różańska A. (2024). Healthcare-Associated Infections and Prevention Programs in General Nursing versus Residential Homes—Results of the Point Prevalence Survey in Polish Long-Term Care Facilities. Medicina.

[27] Economou A. (2025). Public Perceptions on the Efficiency of National Healthcare Systems Before and After the COVID-19 Pandemic. Healthcare.

[28] Audet L.A., Bourgault P., Rochefort C.M. (2018). Associations between nurse education and experience and the risk of mortality and adverse events in acute care hospitals: A systematic review of observational studies. Int. J. Nurs. Stud..

[29] Ichim D.L., Duceac L.D., Marcu C., Iordache A.C., Ciomaga I.C., Luca A.C., Mitrea G., Goroftei E.R.B., Stafie L. (2019). Synthesis and Characterization of Colistin Intercalated Nanoparticles Used to Combat Multi-Drug Resistant Microorganisms. Rev. Chim..

[30] Marczewski K.P., Piegza M., Gospodarczyk N.J., Gospodarczyk A.Z., Marcinek M., Tkocz M., Sosada K. (2024). Evaluation of selected factors influencing sleep disorders in paramedics during the COVID-19 pandemic. Arch. Med. Sci..

[31] Teixeira J., Reis N., Chawłowska E., Rocha P., Czech-Szczapa B., Godinho A.C., Bączyk G., Agrelos J., Jaracz K., Fontoura C. (2025). Current Approaches on Nurse-Performed Interventions to Prevent Healthcare-Acquired Infections: An Umbrella Review. Microorganisms.

[32] Hirani R., Podder D., Stala O., Mohebpour R., Tiwari R.K., Etienne M. (2025). Strategies to Reduce Hospital Length of Stay: Evidence and Challenges. Medicina.

[33] Fattori A., Comotti A., Mazzaracca S., Consonni D., Bordini L., Colombo E., Brambilla P., Bonzini M. (2023). Long-Term Trajectory and Risk Factors of Healthcare Workers’ Mental Health during COVID-19 Pandemic: A 24 Month Longitudinal Cohort Study. Int. J. Environ. Res. Public Health.

[34] Garcia C.d.L., Abreu L.C.d., Ramos J.L.S., Castro C.F.D.d., Smiderle F.R.N., Santos J.A.d., Bezerra I.M.P. (2019). Influence of Burnout on Patient Safety: Systematic Review and Meta-Analysis. Medicina.

[35] Malheiro R., Gomes A.A., Fernandes C., Fareleira A., Lebre A., Pascoalinho D., Gonçalves-Pereira J., Paiva J.-A., Sá-Machado R. (2024). Hospital Context Determinants of Variability in Healthcare-Associated Infection Prevalence: Multi-Level Analysis. Microorganisms.

[36] Todo Bom L.F.P., Mata E.S.F., Cunha H.M.P., Marquês M.d.C.M.P., Dixe M.d.A. (2025). Effectiveness of Nursing Interventions on Preventing the Risk of Infection in Adult Inpatients: Protocol for a Systematic Review. Nurs. Rep..

[37] Pappa D., Koutelekos I., Evangelou E., Dousis E., Mangoulia P., Gerogianni G., Zartaloudi A., Toulia G., Kelesi M., Margari N. (2023). Investigation of Nurses’ Wellbeing towards Errors in Clinical Practice—The Role of Resilience. Medicina.

[38] Sharma M., Bachani R. (2023). Knowledge Attitude Practice Perceived Barriers for the Compliance of Standard Precautions among Medical Nursing Students in Central India. Int. J. Environ. Res. Public Health.

[39] Antolí-Jover A.M., Gázquez-López M., Brieba-del Río P., Martín-Salvador A., Martínez-García E., Sánchez-García I., Álvarez-Serrano M.A. (2025). Prevalence and Predictors of Work–Life Balance Among Nursing Personnel During the Sixth Wave of the Pandemic: The Role of Stress and Sociodemographic and Work-Related Variables. Behav. Sci..

[40] Huang Y., Wang Z., Li Y., Zhao Z., Wang W., Cai C., Wu X., Liu L., Chen M. (2025). Anxiety and burnout in infectious disease nurses: The role of perceived stress and resilience. BMC Nurs..

[41] Claponea R.M., Iorga M. (2023). The Relationship between Burnout and Wellbeing Using Social Support, Organizational Justice, and Lifelong Learning in Healthcare Specialists from Romania. Medicina.

[42] Peñacoba-Puente C., Gil-Almagro F., García-Hedrera F.J., Carmona-Monge F.J. (2025). From Anxiety to Hopelessness: Examining Influential Psychological Processes Affecting Mental Health Status of Spanish Nurses During the COVID-19 Pandemic. Medicina.

[43] Rozmann N., Fusz K., Macharia J.M., Sipos D., Kivés Z., Kövesdi O., Raposa B. (2025). Occupational Stress and Sleep Quality Among Hungarian Nurses in the Post-COVID Era: A Cross-Sectional Study. Healthcare.

