# Supplementary material for: Healthcare-Associated Infections: Knowledge Score and Awareness Among Nurses in Hospitals from North-East Romania
Source: Healthcare (Basel). 2025 Dec 24;14(1):44. doi: 10.3390/healthcare14010044 (PMC12785713; doi:10.3390/healthcare14010044)
Supplement: Supplementary file 1 [file healthcare-14-00044-s001.zip › healthcare-3899544 Supplementary-final-Appendix Nurses knowledge HAIs questionnaire english version.pdf]

QUESTIONNAIRE TO EVALUATE THE LEVEL OF INFORMATION ON HEALTHCARE-ASSOCIATED INFECTIONS AMONG MEDICAL PROFESSIONALS
# - MEDICAL ASSISTANTS -

We invite you to participate in completing the following questionnaire, with the aim of assessing your knowledge of healthcare-associated infections (HAI). Participation in this questionnaire is voluntary and you can withdraw at any time, and all information is confidential and anonymous. Individual responses will not be displayed publicly.

Informed consent: Date:

I. Demographic data

1. Age category:
1. 18-30
2. 31-40
3. 41-50
4. 51-60
5. 61 and over

2. Gender:
1. Female
2. Male

3. Work experience:
1. Under 5 years
2. Between 5-15
3. Over 15 years

4. Specialization
1. Medical department
2. Surgical ward
3. Chronic diseases
4. Palliative care

II. Level of knowledge

5. How important is the role of the medial assistant in preventing and limiting HAI?
1. To a small extent
2. To a very large extent
3. I don't know how to answer.

6. Do you know the factors that favor the occurrence of IAAM?
1. Yes
2. No

7. On a scale from 1 to 5, how do you rate the influence of the following factors in the occurrence of IAAM?

	Not at all	Little	Moderate	Quite a lot	Very much
1.Factors related to medical and healthcare personnel	1	2	3	4	5
2. Patient-related factors	1	2	3	4	5
3. Factors related to the unit	1	2	3	4	5

8. Regarding personnel, on a scale from 1 to 5, how do you assess in your work that the prevention of HAIs is influenced by:

	Not at all	Little	Moderate	A lot	Very much
1. Multitasking at work	1	2	3	4	5
2. Lack of staff	1	2	3	4	5
3. Professional exhaustion/burnout	1	2	3	4	5
4. Insufficient knowledge on the prevention of HAIs	1	2	3	4	5
5. Ineffective team/lack of teamwork	1	2	3	4	5
6. Poor collaboration with the SPLIAAAM service	1	2	3	4	5
7. Poor staff training	1	2	3	4	5
8. Stressful work environment	1	2	3	4	5
9. Staff health status	1	2	3	4	5

9. In general, would you say your health is:

**1**	**2**	**3**	**4**	**5**
poor	mediocrity	Hello	Very good	excellency

10. Compared to last year, how would you rate your physical and mental health? psychic:

**1**	**2**	**3**	**4**	**5**
Much worse	Something worse	Same	Something better	Much better

11. Regarding health problems, on a scale from 1 to 5, how do you rate their influence on daily activity:

	Not at all	Little	Moderate	A lot	Very much
1. Physical component	1	2	3	4	5
2. The psychological component	1	2	3	4	5
3. Social component	1	2	3	4	5

12. Have you had any hospitalizations during the last 5 years of activity?

**1**	**2**	**3**	**4**	**5**
Very rare	rare	Annual	1-2/year	More than 3-5/year

13. In the last calendar year, how would you rate your level of professional burnout, on a scale from 1 to 5?

**1**	**2**	**3**	**4**	**5**
There are no problems.	Rare fatigue	Frequent fatigue	Exhausted	Very exhausted/burnout

14. How intense have you felt body pain lately?

**1**	**2**	**3**	**4**	**5**
I didn't feel it.	Mild pain	Moderate pain	Severe pain	Very severe pain

15. How much has your work been affected by these problems?

**1**	**2**	**3**	**4**	**5**
Not at all	Little	Moderate	Quite a lot	Very much

16. Do you consider that these health problems may be a risk factor in the occurrence of HAI in patients under care by affecting working conditions, overloading with tasks, etc.?

1. Yes

2.No
3.I don't know

17. How many hours of your work schedule do you estimate you spend with the patient daily?

1	2	3
Half	Less than half	More than half

18. Do you consider that you have enough time to perform your work tasks in a safe manner for the patient?

1. Yes
2.No
3. Sometimes yes

19. Do the multiple documents that must be developed in the patient care process affect the quality of the medical act and patient satisfaction?

1. Yes
2. Probably yes
3.No
4.I don't know

20. Do you consider the digitalization of the medical system appropriate for increasing patient safety and easing work tasks?

1. Yes
2. Probably yes
3.No
4.I don't know

21. In the last calendar year, have you had situations in which lack of knowledge led to actions that could have favored the emergence of IAAM?

1. Yes
2.No
3.I don't know

22. How many internal training courses have you taken on HAI prevention?

1	2	3	4	5
Over 2	2 courses	1 course	None	Individual reading

23. Have you participated in conferences, seminars, congresses on the prevention of HAIs?

1	2	3	4	5
Annual	At least 3 years old	Once	Never	I can't afford it financially.

24. Do you consider it useful to emphasize professional training in this field?

1. Yes
2.No
3.I don't know

25. Looking at the factors related to the patient, on a scale from 1 to 5, how do you assess, in your work, their influence on the occurrence of HAI?

	Not at all	Little	Moderate	A lot	Very much
1. Antibiotic resistance	1	2	3	4	5

2. Prolonged hospitalizations	1	2	3	4	5
3. Invasive diagnostic and treatment procedures	1	2	3	4	5
4. Immunodepression	1	2	3	4	5
5. Abuse of antibiotics, self-medication	1	2	3	4	5

2 6. Related to these factors, on a scale from 1 to 5, how do you assess in the unit where you work in the treatment plan, the patient is prescribed:

	Not	Little	Moderate	A lot	Very much
1. injection	1	2	3	4	5
2. perfusion	1	2	3	4	5
3. PO	1	2	3	4	5
4. Other ways	1	2	3	4	5
5. Invasive investigations	1	2	3	4	5
6.Oxygen therapy	1	2	3	4	5
7. Intubation	1	2	3	4	5

27. Is intravenous catheter monitoring unanimously applied in daily practice?

1.Yes, permanently
2. Flashing
3.No

28. Is monitoring of pressure ulcer complications applied in daily practice?

1. Yes
2.No
3. Not the case

29. As a nurse, do you contribute to the identification of HAIs?

**1**	**2**	**3**	**4**	**5**
Not	To a small extent	Largely	To a very large extent	It is not my responsibility.

30. Do you consider teamwork beneficial in preventing and limiting HAIs?

**1**	**2**	**3**
Not necessarily	To a small extent	Largely

31. Do you think that the team at work is functional, is there teamwork?

1. Yes
2.No
3.I don't know

32. Do you know what standard precautions are?

1. Yes
2.No
3.I don't know

33. On a scale from 1 to 5, what are the risk factors that favor the occurrence of HAIs in the unit where you work?

	Not at all	Little	Moderate	A lot	Very much
1. Insufficient staff	1	2	3	4	5
2. High degree of patient dependency	1	2	3	4	5
3. Burnout/professional burnout	1	2	3	4	5
4. Lack of knowledge regarding the prevention of HAIs	1	2	3	4	5
5. Lack of disinfectant products	1	2	3	4	5

6. Lack of equipment/machinery for cleaning and disinfection	1	2	3	4	5
7. Antibiotherapy without antibiogram/inappropriate	1	2	3	4	5
8. Deficient circuits	1	2	3	4	5
9. Dysfunctions of sterilization of reusable materials	1	2	3	4	5
10. Lack of knowledge regarding cleaning, disinfection and sterilization taking into account the risk level of activity sectors, surfaces, devices and sanitary materials	1	2	3	4	5
11. Overcrowding of wards, wards with more than 4 beds	1	2	3	4	5

34. On a scale from 1 to 5, what are the measures to prevent and limit HAI applied in daily practice in the unit where you work?

	Not at all	Little	Moderate	A lot	Very much
1. Fair and safe practices	1	2	3	4	5
2. Functional circuits according to the regulations in force	1	2	3	4	5
3. Educating/training staff on HAI prevention	1	2	3	4	5
4. Institutional culture focused on strengthening the climate of patient security and safety	1	2	3	4	5
5. Antimicrobial resistance procedure well established and followed	1	2	3	4	5
6. Well-organized sterilization service with clear procedures	1	2	3	4	5
7. Proper hand hygiene	1	2	3	4	5
8. Following standard precautions	1	2	3	4	5
9. Respecting bed occupancy	1	2	3	4	5
10 Sufficient medical and nursing staff	1	2	3	4	5
11. Overcrowding of wards, wards with more than 4 beds	1	2	3	4	5

35. How true or false, on a scale from 1 to 5, where 1 is false and 5 is true, is the following statement for you?

1. IAAM are undervalued	1	2	3	4	5
2. IAAMs, although identified, are not all reported	1	2	3	4	5
3. The main etiological agents of HAI are multidrug-resistant hospital germs	1	2	3	4	5
4. HAI is a major population health problem	1	2	3	4	5
5. Failure to adapt to the HAI surveillance system is a main cause of the lack of identification or late identification of HAI	1	2	3	4	5
6. Knowing and following standard precautions are the main way to prevent and limit HAIs.	1	2	3	4	5
7. Staff training on prevention and containment of HAIs is insufficient	1	2	3	4	5
8. Standard precautions are not known at all levels in the hospital environment	1	2	3	4	5

36. What is the incidence of IAAM in the unit where you work?

**1**	**2**	**3**	**4**	**5**
0-1%	1.1-2%	2.1-3%	3.1-4%	4.1% and above

37. In the unit where you work, do you have an effective collaboration with the department for prevention and limitation of HAIs (does it provide you with clear, efficient information, procedures, and work instructions)?

1	2	3
Not	To a small extent	Largely

3 8. Do you consider the role of SPLIAAM important in the process of preventing and limiting IAAM?

1	2	3
Not	To a small extent	Largely

39. Do you consider the role of the care director important in the process of preventing and limiting HAIs?

1	2	3
Not	To a small extent	Largely

40. In the prevention and ongoing surveillance of HAI, on a scale of 1 to 5, how do you apply the following interventions:

	Not at all	Little	Moderate	A lot	Very much
1. Assessment of the patient's infectious risk upon admission	1	2	3	4	5
2. Choosing investigation and treatment procedures with minimal risk of HAI	1	2	3	4	5
3. Compliance with procedures, protocols and work instructions regarding asepsis and antisepsis	1	2	3	4	5
4. Intensive hygiene and control programs	1	2	3	4	5
5. Empowering staff, clear work tasks	1	2	3	4	5
6. Permanent monitoring of central and peripheral venous catheterization	1	2	3	4	5
7. Screening of patients upon admission	1	2	3	4	5
8. Isolation of sources of infection	1	2	3	4	5
9. Appropriate functional circuits	1	2	3	4	5
10 Sufficient medical and nursing staff	1	2	3	4	5
11. Overcrowding of wards, wards with more than 4 beds	1	2	3	4	5
12. Processing and distributing food under optimal hygienic conditions	1	2	3	4	5
13. Periodic water control	1	2	3	4	5
14. Respect for the correct management of medical and household waste	1	2	3	4	5
15. Sterilization service according to current standards	1	2	3	4	5
16. Hand hygiene	1	2	3	4	5
17. Permanent monitoring of patients regarding the occurrence of HAIs	1	2	3	4	5
18. Nominal reporting of the case identified with IAAM	1	2	3	4	5
19. Urgently institute related treatment	1	2	3	4	5
20. Isolation, if necessary, of the patient	1	2	3	4	5
21. Justified administration of antibiotics	1	2	3	4	5
22. Good interdisciplinary collaboration (epidemiologist, attending physician, laboratory, SPLIAA, unit	1	2	3	4	5

management)					
23. - Training of medical and healthcare personnel for the application of procedures for the prevention and limitation of HAI	1	2	3	4	5

41. Is epidemiological triage of personnel carried out in the unit where you will be working?

1. Yes
2.No

42. Is epidemiological triage of patients performed in the unit where you will be working?

1. Yes
2.No

III. Feedback on completing the questionnaire

43. Do you believe that participating in this study will bring benefits, new information regarding the importance of preventing and limiting HAIs?

1	2	3
Not	To a small extent	Largely

44. To what extent does participation in the study contribute to increasing patient safety through the information received?

1	2	3
Does not contribute	To a small extent	Largely

45. To what extent does participation in the study contribute to increasing the identification and reporting of HAIs through the information received?

1	2	3
Does not contribute	To a small extent	Largely
