# Supplementary material for: Healthcare-Associated Infections: Knowledge Score and Awareness Among Nurses in Hospitals from North-East Romania
Source: Healthcare (Basel). 2025 Dec 24;14(1):44. doi: 10.3390/healthcare14010044 (PMC12785713; doi:10.3390/healthcare14010044)
Supplement: Supplementary file 1 [file healthcare-14-00044-s001.zip › healthcare-3899544 Supplementary-final.pdf]

Supplementary Material

Model 6 has led to the following equation from which the knowledge score values could be estimated in relation to the awareness of the role and professional experience of the nurse in preventing and limiting HAIs. (Table S1).

$$Y\ score1 = 29.60 + 1.87\ R1 + 2.72\ R2 + 0{,}38\ R3 + 3.69\ unit + 3.92\ personnel + 1.50\ patient \quad (1)$$

Table S1. Multivariate linear regression. Regression line coefficients. Model 6 of the role and nurses professional experience in preventing and limiting HAIs.

Model 6*	Non–standardised coefficients		*t*	Sig.	95% Confidence Interval for B	
	B	Standard error			-95% CI	+95% CI
(Constant)	29.604	5.897	5.020	0.001	17.996	41.212
R1	1.872	1.438	1.301	0.194	-0.960	4.703
R2	2.719	0.827	3.286	0.001	1.090	4.347
R3	0.381	1.326	0.287	0.774	-2.230	2.992
unit	3687	0.908	4.061	0.001	1.900	5.474
personnel	3.924	0.909	4.316	0.001	2.134	5.713
patient	1.496	0.719	2.080	0.038	0.080	2.912

* Dependent variable: Score1.

Model 9 has led to the following equation from which the knowledge score values could be estimated in relation to the appreciation of factors favoring the occurrence of HAIs (Table S2).

$$Yscore2 = 45.01 + 1.50\ P1 + 1.36\ P2 + 1.68\ P3 + 1.31\ P4 + 1.60\ P5 + 0.51\ P6 + 0.70\ P7 + 1.93\ P8 + 1.60P9 \quad (2)$$

Table S2. Multivariate linear regression. Regression line coefficients. Model 9 assessment of factors favoring the occurrence of HAIs.

Model 9*	Non–standardised coefficients		*t*	Sig.	95% Confidence Interval for B	
	B	Standard error			-95% CI	+95% CI
(Constant)	45.009	1.257	35.810	0.001	42.535	47.483
P1	1.498	0.383	3.907	0.001	0.743	2.252
P2	1.357	0.462	2.934	0.004	0.447	2.267
P3	1.679	0.399	4.203	0.001	0.893	2.465
P4	1.311	0.418	3.138	0.002	0.488	2.133
P5	1.593	0.407	3.912	0.001	0.792	2.395
P6	0.539	0.426	1.265	0.207	-0.300	1.379
P7	0.704	0.446	1.577	0.116	-0.175	1.582
P8	1.926	0.413	4.667	0.001	1.114	2.739
P9	1.601	0.452	3.538	0.001	0.710	2.492

* Dependent variable: Score2.

Model 6, statistically significant, has led to the following equation from which the values of the knowledge score in relation to personal problems in relation to daily activity that could influence the prevention of HAIs could be estimated (Table S3).

$$Y\ score3 = 49.36 + 2.41\ S1 + 1.14\ S2 + 3.95\ S3 + 1.79\ S4 + 1.68\ S5 + 1.15\ S6 \quad (3)$$

Table S3. Multivariate linear regression. Regression line coefficients. Model 9 assessment of factors favoring the occurrence of HAIs.

Model 6*.	Non–standardised coefficients		*t*	Sig.	95% Confidence Interval for B	
	B	Standard error			-95% CI	+95% CI
(Constant)	49.360	3.355	14.712	0.000	42.755	55.964
S1	2.405	0.769	3.127	0.002	0.891	3.919
S2	1.139	0.698	1.632	0.104	-0.235	2.513
S3	3.946	0.827	4.772	0.000	2.318	5.573
S4	1.793	0.770	2.328	0.021	0.277	3.309
S5	1.675	0.747	2.241	0.026	0.203	3.146
S6	1.150	0.435	2.646	0.009	0.294	2.006

* Dependent variable: Score3.

Table S4. Correlation between the knowledge score of prevention and limitation of HAIs and the answers regarding the level of knowledge in prevention and limitation of HAIs.

Items / Level of knowledge in preventing and limiting HAIs	Low score (n=65)		Moderate score (n=156)		High score (n=67)		Chi² test (*p*)
	n	%	n	%	n	%	
Nurse role							
not at all	3	4.6	0	0,0	0	0.0	0.039
very much	57	87.7	141	90.4	65	97.0	
Profesional experience							
not at all	5	7.7	0	0,0	0	0.0	0.001
very much	46	70.8	119	76.3	59	88.1	
Profesional training							
not at all	2	3.1	0	0,0	0	0.0	0.007
very much	56	86.2	130	83.3	64	95.5	
Factors related to the unit							
not at all	2	3.1	0	0,0	0	0,0	0.001
very much	5	7.7	44	28.2	35	52.2	
Factors related to the staff							
not at all	2	3.1	0	0.0	0	0.0	0.001
very much	8	12.3	67	42.9	46	68.7	
Factors related to the patient							
not at all	2	3,1	0	0.0	0	0.0	0.001
very much	7	10.8	39	25.0	33	49.3	
Lack of medical staff							
not at all	7	10.8	3	1.9	1	1.5	0.001
very much	4	6.2	47	30.1	41	61.2	
Burnout							
not at all	7	10.8	0	0.0	0	0.0	0.001
foarte mult	4	6.2	37	23.7	50	74.6	
Insufficient knowledge							
not at all	13	20.0	4	2,6	1	1.5	0.001
very much	1	1.5	43	27.6	49	73.1	
Inefficient team							0.001

not at all	17	26.2	1	0.6	0	0.0	
very much	0	0.0	64	19.9	46	68.7	
Poor collaboration with the HAIs Dept. of Prevention							
not at all	25	38.5	8	5,1	0	0.0	0.001
very much	1	1.5	26	16.7	42	62.7	
Poor staff training							
not at all	23	35.4	6	3.8	0	0.0	0.001
very much	1	1.5	35	22.4	46	68.7	
Stressful work environment							
not at all	11	16.9	0	0.0	0	0.0	0.001
very much	0	0.0	27	17.3	51	76.1	
Staff health status							
not at all	17	26.2	2	1.3	0	0.0	0.001
very much	0	0.0	27	17.3	53	79.1	
Multiple work tasks							
not at all	10	15.4	0	0.0	0	0.0	0.001
very much	3	4.6	63	40.4	55	82.1	

Table S5. Correlation between the knowledge score of HAIs prevention and limitation and personal problems that can influence the prevention of HAIs.

**Items / Personal problems**	**Low score (n=65)**		**Moderate score (n=156)**		**High score (n=67)**		**Chi^2 test (*p*)**
	**n**	**%**	**n**	**%**	**n**	**%**	
Health status							
poor	4	6.2	4	2.6	4	6.0	**0.031**
good	49	75.4	109	69.9	41	61.2	
excellent	3	4.6	6	3.8	6	9.0	
Health status compared to last year							
much worse	2	3.1	4	2.6	2	3.0	**0.032**
the same	49	75.4	94	60.3	35	52.2	
much better	2	3.1	13	8.3	7	10.4	
Physical component							
not at all	14	21.5	5	3.2	0	0,0	**0.001**
very much	2	3.1	26	16.7	40	59.7	
Psychological component							
not at all	18	27.7	8	5.1	1	1.5	**0.001**
very much	5	7.7	49	31.4	49	73.1	
Social component							
not at all	24	36.9	9	5.8	1	1.5	**0.001**
very much	2	3.1	17	10.9	28	41.8	
Hospitalizations in the last 5 years							
3-4 per year	15	23.1	29	18.6	13	19.4	0.970
1-2 per year	5	7.7	9	5.8	4	6.0	
annually	4	6.2	11	7.1	6	9.0	
rarely	41	63.1	107	68.6	44	65.7	
Professional burnout							
very exhausted	3	4.6	17	10.9	7	10.4	0.379
exhausted	10	15.4	22	14.1	4	6.0	
frequent fatigue	25	38.5	63	40.4	34	50.7	

rare fatigue	20	30.8	42	26.9	18	26.9	
no problems	7	10.8	12	7.7	4	6.0	
Recent pain							
severe	39	60.0	91	58.3	32	47.8	0.546
mild	20	30.8	54	34.6	28	41.8	
no pain	6	9.2	11	7.1	7	10.4	
Work impact							
very much	0	0.0	7	4.5	2	3.0	**0.034**
not at all	15	23.1	30	19.2	24	35.8	
Health problems							
Yes	14	21.5	58	37.2	28	41.8	**0.004**
No	47	72.3	26	16.7	9	13.4	
